# Impact of treated wastewater on the mechanical properties and durability of concrete

**DOI:** 10.1038/s41598-026-52561-0

**Published:** 2026-05-19

**Authors:** Omar Abdelazim, Ibrahim Abdel-Latif, Sayed Ismail, Mohamed A. Khalaf

**Affiliations:** 1 Structural Engineering Department, Faculty of Engineering, Ain Shams University, Cairo, Egypt; 2 Public Works Department, Faculty of Engineering, Ain Shams University, Cairo, Egypt

**Keywords:** Treated wastewater, Mechanical properties, Durability, Recycling, Sustainability, Engineering, Environmental sciences, Materials science

## Abstract

Recently, the world has been facing a crisis in water demand. Therefore, countries started to focus on wastewater treatment to address the shortage of freshwater. Since the concrete industry consumes vast amounts of water, researchers started investigating the incorporation of treated wastewater (TWW) in concrete instead of potable water. However, the impact of variations in the properties of TWW on the durability of concrete is still underexplored. This study investigates the influence of TWW on the mechanical properties and durability of concrete. Two secondary treated TWW samples were collected from different sources. The effect of using TWW for mixing and curing of concrete was evaluated through slump, compressive strength, modulus of elasticity, split tensile strength, water absorption, water penetration resistance, rapid chloride penetration, sorptivity, accelerated corrosion, and SEM analysis. Results showed that using TWW caused a reduction in compressive strength at early ages, while the reduction became negligible at later ages. Moreover, the impact of TWW on the durability was not significant, except for accelerated corrosion results as the mass loss was significantly higher compared to OPC. Moreover, variations in TWW properties affected its performance, as TWW with higher TDS showed better performance, due to the pore filling effect.

## Introduction

Freshwater is a scarce resource, as fresh water represents only 2.4% of the total water on earth. Moreover, 68.7% fresh water exists in glaciers and ice caps and 30.1% is groundwater, leaving just 1.2% is in rivers and lakes^1^. This limited availability makes freshwater scarcity a significant global issue. By 2030, 1.6 billion people are expected to lack safely managed drinking water^2^. The United Nations’ World Water Assessment Program reported that 56% of freshwater withdrawals are released as wastewater, with 80% of the released wastewater is discharged without proper treatment^3^. Egypt is one of the countries affected by the scarcity of freshwater. Therefore, Egypt started to focus on developing the field of wastewater treatment and reuse. In 2014 the total number of wastewater treatment plants was 300 plants, in 2024 the number of wastewater treatment plants reached 588, with a capacity of 18.82 million m^3^ per day. Treated wastewater (TWW) is used in a wide range of applications, which can be divided into three main categories: Irrigation, Civil purposes and Industrial applications^4^. Recently, researchers started to study the suitability of using treated wastewater in the concrete industry, as the concrete industry is one of the biggest industries in the world with annual production that reached 14 billion m^3^ of concrete in 2020^5^. Moreover, producing 1 m^3^ of concrete consumes about 150 L of water, and if the wasted water is taken into consideration, the amount of water involved in producing 1 m^3^ of concrete can reach 500 L^6^. TWW represents great potential in facing the water crisis if it’s proved to be suitable for use in concrete industry, either in mixing or curing of concrete. Several studies investigated the influence of using TWW in concrete^7,8^. However, there are some contradictions in the literature regarding the effect of TWW on the properties of concrete^9,10^. While using TWW in mixing of concrete was reported to increase the compressive strength^8,11^. Other studies showed that using TWW as mixing water leads to a reduction in compressive strength^12,13^. Almeida M and Tonetti A^14^, suggested that the diversity in the effect of TWW on the properties of concrete is due to the wide variation of wastewater properties and its different quality parameters. Furthermore, they added that although the majority of research found negative effects of using TWW on the compressive strength of concrete, most of the results were accepted, as the reduction didn’t exceed 10%^14^. However, a clear understanding of how variations in treated wastewater properties influence both the mechanical and durability performance of concrete remains limited. This study investigates the effect of incorporation of TWW as mixing and curing water, on the mechanical properties and durability of concrete. The study aims to enrich our understanding on how the variations in the properties of TWW can impact the concrete properties, ultimately contributing to the development of more sustainable practices in construction.

## Materials and mix proportions

In this study, three types of water were collected from different sources. potable water (PW) collected from a local source in Cairo was used in mixing water for the control sample. Additionally, treated wastewater samples were collected from two wastewater treatment plants, sourced from environments with varying characteristics. This diversity was crucial for assessing how variations in water quality affect the properties of concrete when used as mixing or curing water. TWW1 was collected from a wastewater treatment plant in Cairo, the wastewater was subjected to a secondary treatment system. TWW2 was collected from a wastewater treatment plant in Suez, where the wastewater undergoes a secondary treatment system. Table 1 summarizes the chemical properties of the water samples. Moreover, ordinary Portland cement CEM I 42.5 N was used as the main binder in the concrete mix. Crushed coarse and fine aggregates were collected from a source in Cairo with specific gravity of 2.61 and 2.6 respectively. The nominal maximum size of the coarse aggregates was 20 mm. Three mixes were developed in this study to investigate the influence of treated wastewater on the properties of concrete. The control mix utilized PW as the mixing water, while in the other two mixes. PW was fully replaced with TWW1 and TWW2 respectively. Moreover, the concrete mix was designed so that the control mix has compressive strength not less than 25 MPa at 28 days. The mix design used in this study is presented in Table 2.

**Table 1:** Chemical properties of water samples.

Test	Unit	PW	TWW1	TWW2
pH		7	7.6	7.8
Sodium sulphide	mg/L	0.01	0.01	0.01
Carbonates & bicarbonates	mg/L	222	260	296
Organic matter	mg/L	5.4	6.1	3.8
C.O.D	mg/L	2.4	3.1	4.3
Turbidity	mg/L	0.241	0.626	8.76
T.D.S	mg/L	221	479	1655
T.S.S	mg/L	0.6	0.85	0.7
T.S	mg/L	229	486	1774
Chlorides	mg/L	24.9	97.6	*552*
Sulphates (SO_4_)	mg/L	54.7	74.2	266

**Table 2:** Mix proportions needed to produce 1 m^3^of concrete.

	Cement	Water	Sand	Crushed stone
Weight (Kg)	340	190	605	1225
% by weight	1	0.56	1.78	3.6

### Test procedures

**Fig. 1:**
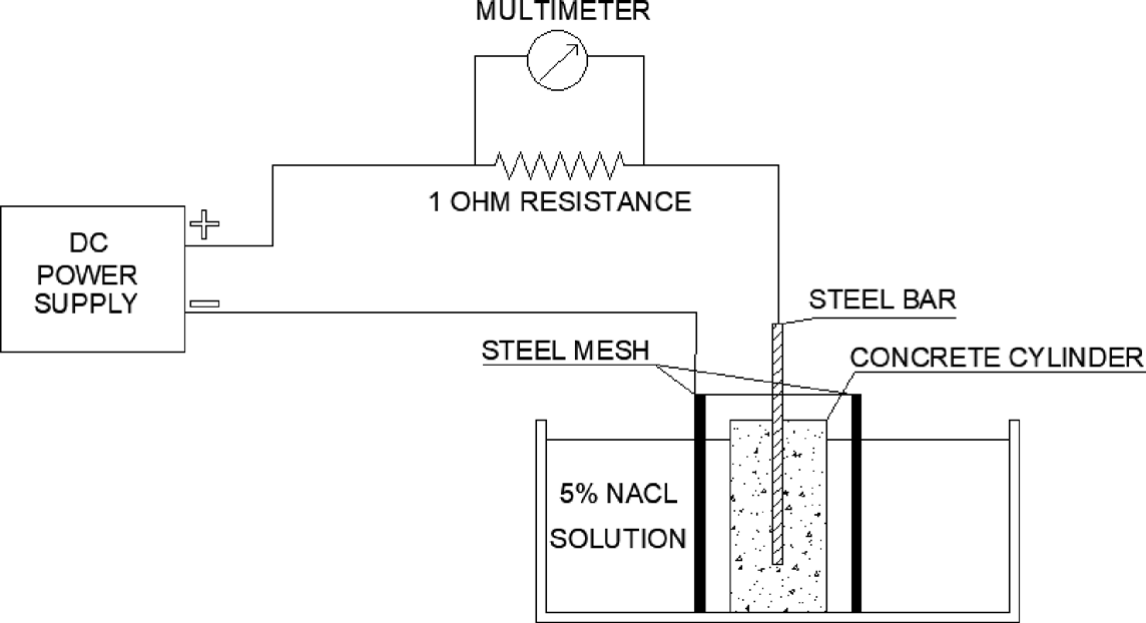
Schematic drawing of accelerated corrosion test setup

Slump test was carried out according to ASTM C143^15^ to evaluate the fresh properties of concrete. Compressive strength test was carried out according to BS-EN 12,390^16^, cubes with 10 × 10 × 10 cm dimensions were casted and tested in compression at 3, 7, 28, 60 and 90 days respectively. Cubes from each mix were divided into 2 groups, one group was cured in PW - Normal treatment (N.T), the other group was cured in TWW1 – Wastewater treatment (W.W.T). This was meant to study the effect of treated wastewater as curing water on the compressive strength of concrete. Modulus of elasticity test was evaluated based on ASTM 469^17^. Splitting tensile strength was performed following ASTM C496^18^ on 10 × 20 cm cylinders. Water absorption test was conducted in accordance with BS 1881 Part 122^19^, Moreover, Water penetration resistance was assessed in accordance with DIN 1048^20^ on 15 × 15 × 15 cm cubes. Rapid chloride penetration test was performed according to ASTM C1202^21^. Additionally, Sorptivity test was carried out according to ASTM C1585^22^. Accelerated corrosion test was performed to determine the effect of treated wastewater on the corrosion of steel bars embedded in concrete. Hence, 10 × 20 cm cylinders were cast with a 10 mm steel bar embedded in its center, with 5 cm clear cover from each side. DC power supply that provides 12 volts was used and the specimens were placed in a NaCL solution with 5% concentration^23^, as shown in Fig. 1. Steel mesh was also placed in the solution and connected to the negative terminal to act as cathode^24^. The cylinders were then split in half after the test and the bar was extracted from the specimen. Furthermore, the corrosion products were removed from the steel bar. Finally, the bar was weighted to measure the % of mass loss. All results represent the average of three test specimens.

## Results and discussion

### Slump

**Fig. 2:**
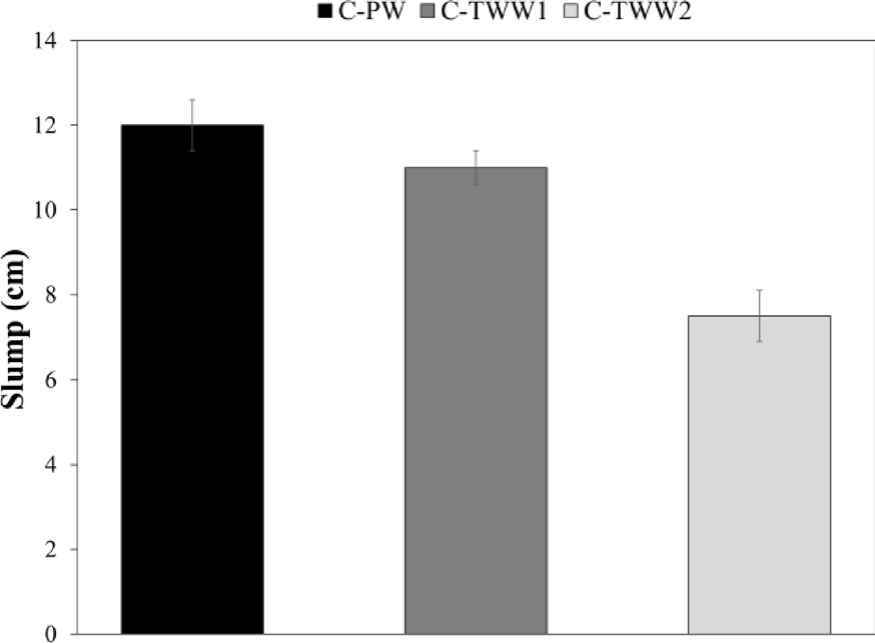
Slump test results.

Figure 2 presents the results of the slump test, which show that using treated wastewater decreases the slump value. The obtained results comply with the results found by previous researchers in the literature^7,8,25^. The decrease in the slump values can be attributed to the high amounts of solids present in TWW, as it can decrease the actual amount of water^7,25^.The proposed explanation for the reduction in slump value can be supported by the T.S content results of the water samples, where the T.S content for PW, TWW1 and TWW2 were 229, 486, and 1774 mg/l respectively. It can be noticed that C-TWW2 which contained the highest T.S content, showed the lowest slump value.

### Compressive strength

**Fig. 3:**
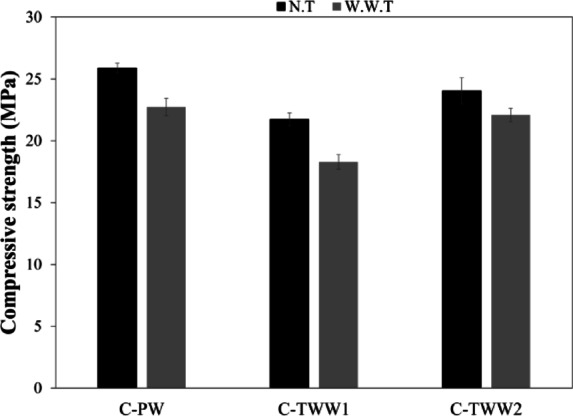
28 days compressive strength results.

Table 3 show the compressive strength test results at different ages. It can be noticed that at early ages, up to 28 days, the compressive strength of specimens mixed with TWW showed a reduction in compressive strength, while C-TWW1 showed the lowest compressive strength value at 28 days with 16% reduction. C-TWW2 showed a slight reduction in compressive strength at 28 days, with only 7% reduction in compressive strength compared to the control sample. Figure 3 shows the 28-day compressive strength results cured in PW and TWW. It was observed from the results that at early ages, curing in treated wastewater reduced compressive strength with a percentage that ranged between 7.6 and 13.3%. However, at late ages, the reduction in the compressive strength decreased in all treated wastewater mixes. Although C-TWW1 still had compressive strength lower than C-TWW2, the reduction in compressive strength in both mixes was nearly negligible, as the reduction was 1.2 and 0.9% for C-TWW1 and C-TWW2 respectively. Furthermore, the reduction in compressive strength due to curing in TWW1 also decreased to a maximum of 4.4% with C-TWW1. The obtained results comply with the findings of previous researchers that using TWW in concrete mixing decrease the compressive strength^12,26–28^.

The obtained results can be attributed to several factors. The decrease in compressive strength can be due to the presence of chlorides in the TWW, as chlorides are reported to decrease the compressive strength^29^. Moreover, the presence of fine solids can lead to a pore filling effect which can reduce the porosity and thus increase the compressive strength^30^. It is suggested that there is a dual effect that happened between the reduction in compressive strength due to chlorides and the enhancement in the compressive strength due to pore filling effect. That explains that although C-TWW2 had high chloride content, the negative effect was reduced due to the high T.S content. Therefore, the reduction is compressive strength of C-TWW2 was less significant. The enhancement of compressive strength at late ages can be explained by the presence of sulphates, as it was reported that sulphates cause a reduction in compressive strength that is diminished at late ages^29^. Furthermore, the enhancement in compressive strength can be due to the existence of a type of bacteria in TWW, which leads to the precipitation of CaCo_3_ leading to the filling of pores. Hence, it eventually leads to the increase of compressive strength, this process is known as “self-healing process”^31^.

**Table 3:** Compressive strength test results at different ages (MPa).

Age (days)	3	7	28	60	90
C-PW (N.T)	7.65	17.44	25.87	28.83	29.15
C-PW (W.W.T)	7.85	16.88	22.73	27.83	28.24
C-TWW1 (N.T)	8.61	12.43	21.73	22.10	28.80
C-TWW1 (W.W.T)	7.93	11.15	18.29	21.86	28.57
C-TWW2 (N.T)	12.43	19.90	24.05	28.80	28.89
C-TWW2 (W.W.T)	8.54	16.05	22.08	26.62	27.86

### Modulus of elasticity

**Fig. 4:**
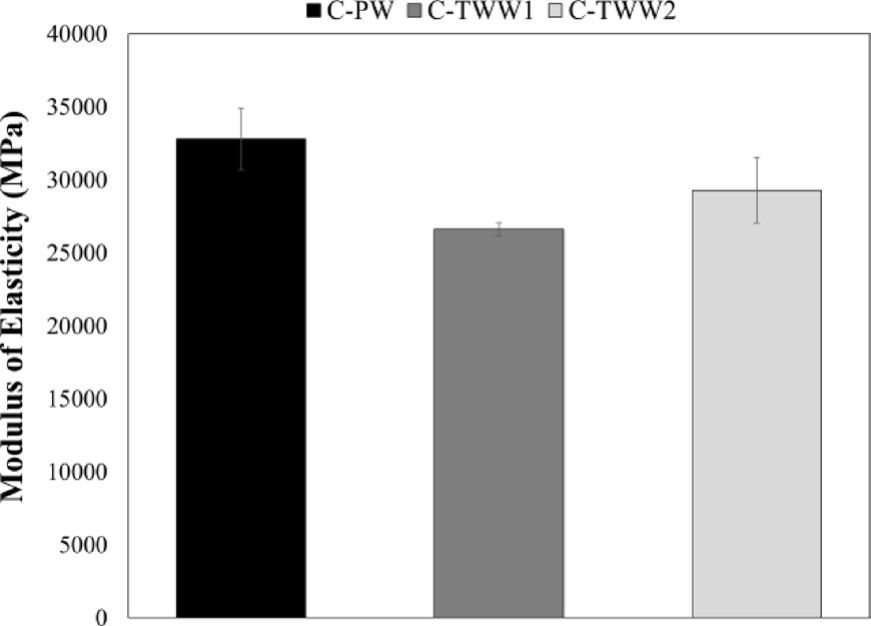
Modulus of elasticity test results.

Figure 4 presents the results of the modulus of elasticity test, as it was carried out according to ASTM C469^18^. Results show that C-TWW1 had the lowest modulus of elasticity, with a reduction of 18.85% compared to the control sample. While C-TWW2 had a reduction of 10.7% in the modulus of elasticity. Results comply with the findings from the compressive strength test.

### Splitting tensile strength

**Fig. 5:**
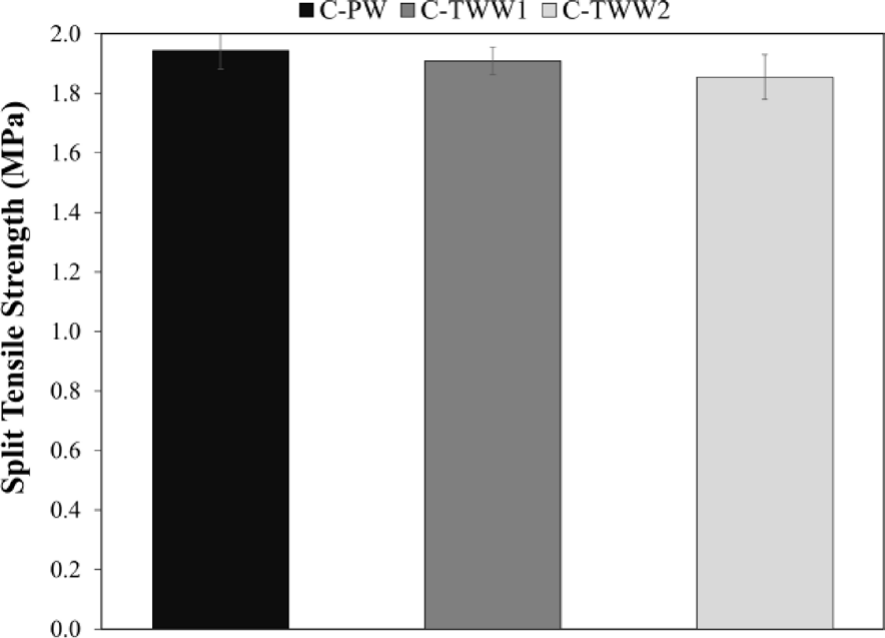
Split tensile strength test results.

Figure 5 displays the splitting tensile strength test results, it was found that using TWW in mixing of concrete causes a reduction in the split tensile strength. However, the reduction in the splitting tensile strength due to mixing with TWW is not significant. As the reduction for C-TWW1 and C-TWW2 was 1.77 and 4.59% respectively. These results comply with the previous literature findings, that the TWW causes a slight reduction in the splitting tensile strength^9,14,32^.

### Water absorption

**Fig. 6:**
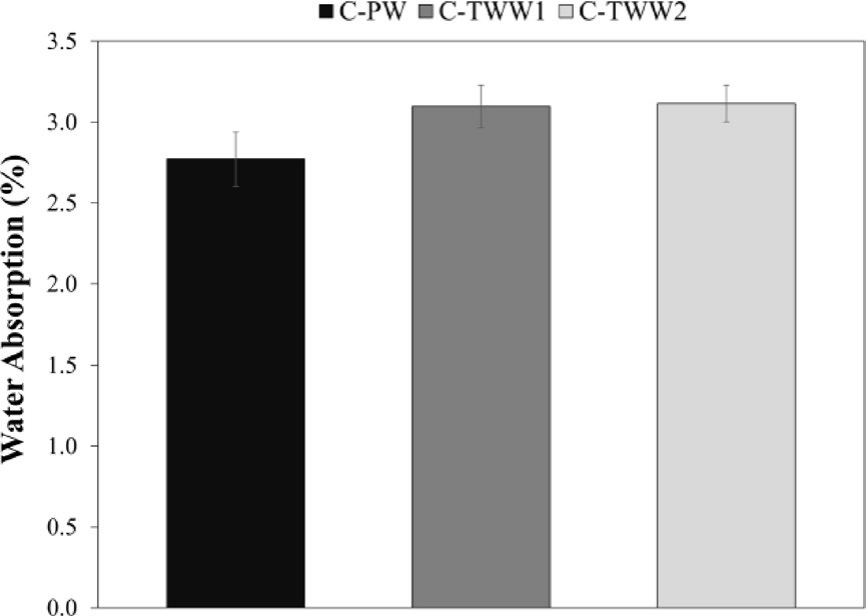
Water absorption test results.

Results for water absorption test show that using TWW increase the % of water absorption, as both C-TWW1 and C-TWW2 showed higher water absorption than the control sample as shown in Fig. 6. However, the increase in water absorption was not significant as the % of increase in water absorption was 11.83 and 12.41% for C-TWW1 and C-TWW2 respectively. These results comply with the findings of the previous researchers, as some researchers stated that the increase in water absorption % was not significant^11,27^.

### Water penetration resistance

**Fig. 7:**
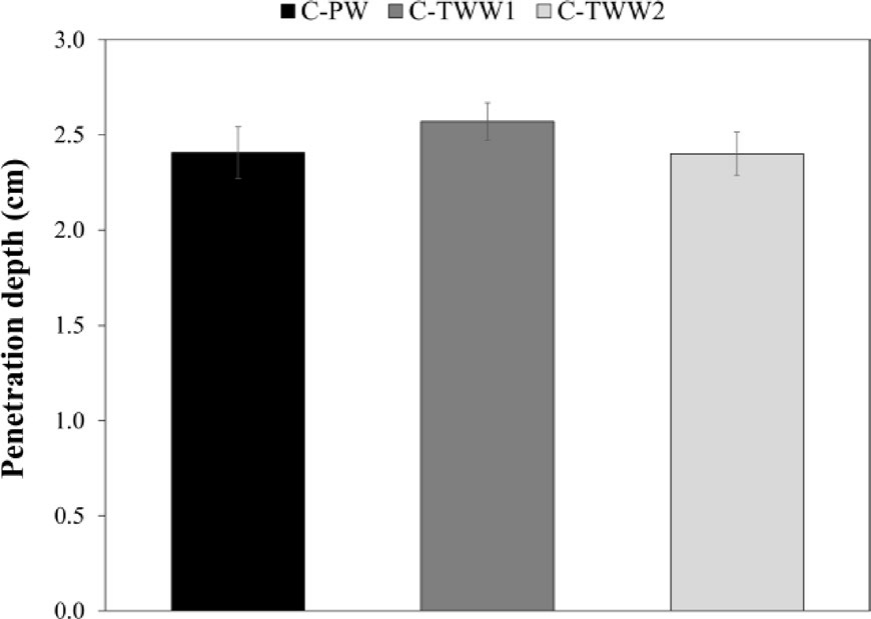
Water penetration resistance test results.

Water penetration resistance test was performed according to DIN-1048-Part 5^20^. The tested cubes were split in half, and the penetration depth was measured at 5 points, and the average of the reading is calculated. Figure 7 show the results of the test. It can be observed that the effect of TWW on water penetration resistance is not significant, since C-TWW2 approximately had the same penetration depth as the control sample, while C-TWW1 showed a slight increase in the penetration depth, as the penetration depth increased by only 1.6 mm. The obtained results comply with the previous findings in the literature, as some researchers stated that TWW had a negative effect on water penetration resistance, but the effect is not significant^27^.

### Rapid chloride penetration

**Fig. 8:**
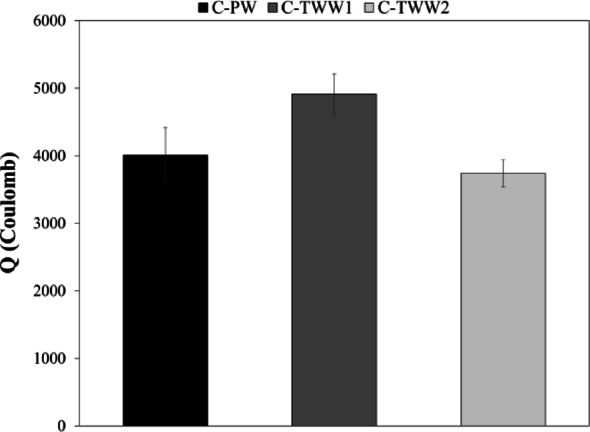
Rapid chloride penetration results.

Rapid chloride penetration test was carried out according to ASTM C1202^21^. Charge Q in coulombs was then calculated as shown in Fig. 8. It can be seen from the test results that C-TWW1 had the highest chloride ion permeability, while C-TWW2 displayed slightly better chloride ion permeability compared to the control sample. Results comply with the findings of this research regarding the effect of TWW on the mechanical properties. where the negative effects of TWW were higher in C-TWW1 than C-TWW2. The enhancement in the results regarding C-TWW2 complies with the findings of previous researchers, where they stated that using TWW in mixing can enhance the chloride ion permeability^13^. This can be attributed to the pore filling effect due to the high amounts of fine solids in TWW2.

### Sorptivity

**Fig. 9:**
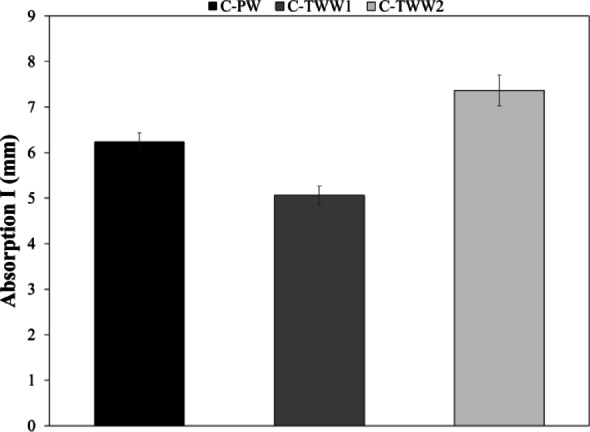
Capillary water absorption I (mm).

Sorptivity test results show that C-TWW1 had the lowest absorption due to capillary action, while C-TWW2 had the highest absorption compared to the control sample as shown in Fig. 9. However, results of the sorptivity test alone can be misleading, as results can be understood as if C-TWW1 have the lowest porosity. However, from the other durability tests, it can be concluded that C-TWW1 had the lowest amount of capillary pores. Pores with size larger or smaller than the capillary pores will fail to absorb water due to capillary action. In other terms, total porosity is different than capillary pores. From the test results, it can be said that C-TWW1 had the lowest amount of capillary pores while C-TWW2 had the highest amount of capillary pores.

### Accelerated corrosion

**Fig. 10:**
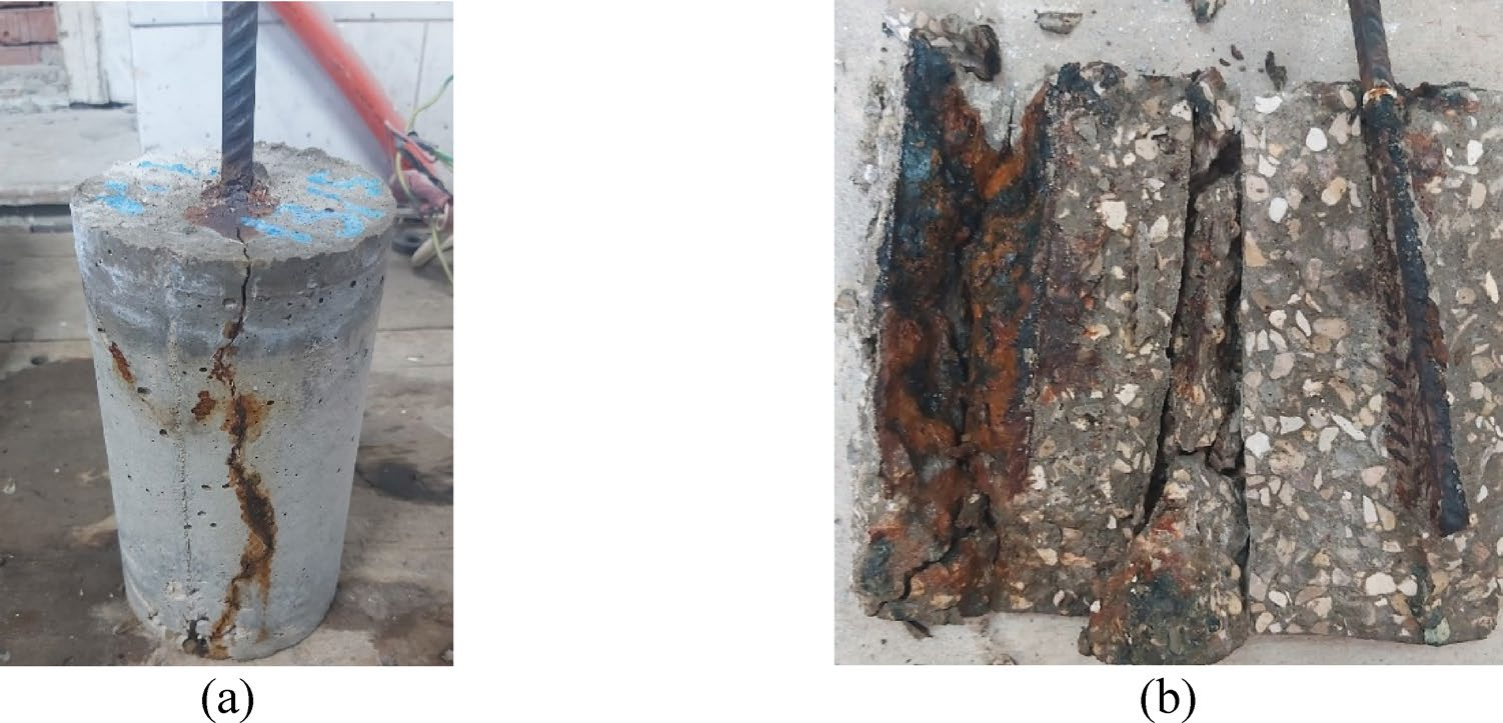
Accelerated corrosion specimens after test (**a**) Longitudinal crack, (**b**) Corrosion of steel bar.

Accelerated corrosion test was carried out for a period of 7 days. Cracks were noticed to appear on the surface of the specimens after 65 h. After 7 days the specimens were removed, it was noticed that there were cracks along the length of specimens, as shown in Fig. 10a. Specimens were then split in half, and the steel bars were extracted from the specimens as shown in Fig. 10b. It was observed that severe corrosion had taken place in the steel rebars, it was also noticed that the corrosion wasn’t completely uniform, and pitting corrosion was noticed on the steel bars after the corrosion products were removed from the tested bars.

**Fig. 11:**
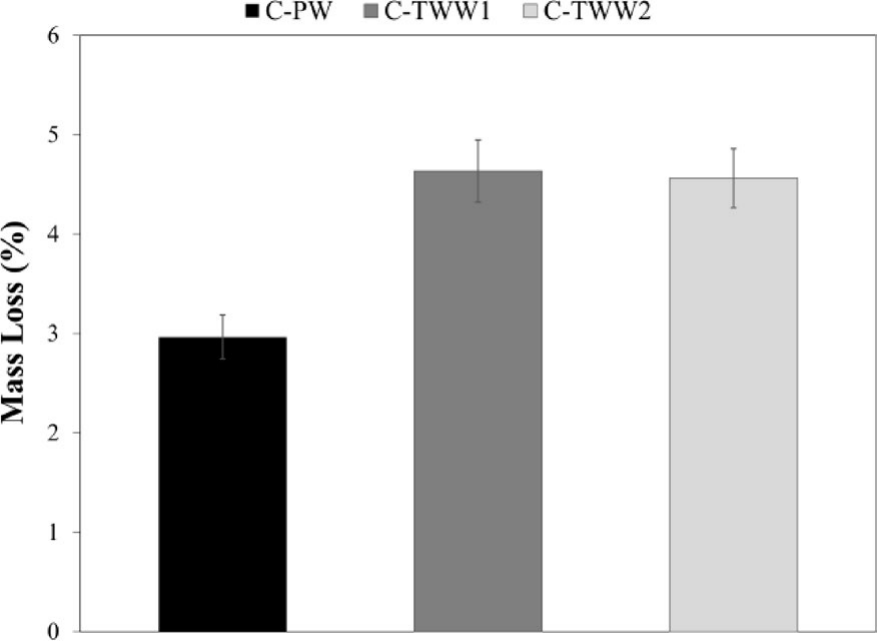
Mass loss in steel bars.

The percentage of mass loss was calculated as shown in Fig. 11. Results indicate that C-TWW1 and C-TWW2 experienced more severe corrosion than the control sample (C-PW), with mass loss values of 2.97%, 4.63%, and 4.56% for C-PW, C-TWW1, and C-TWW2, respectively. It can be observed that TWW increases corrosion susceptibility. Although C-TWW2 had a higher chloride content (552 mg/L) than C-TWW1, the difference in mass loss between the two mixes was negligible, suggesting that chloride content is not the only factor influencing corrosion behavior. According to ASTM C1602, the chloride content of mixing water should not exceed 1000 mg/L for reinforced concrete applications, indicating that both TWW samples were within acceptable limits. These observations suggest that, despite complying with ASTM C1602, the use of TWW may still contribute to increased corrosion and reinforced concrete. Other factors, such as total dissolved solids (TDS), can increase the electrical conductivity of concrete, disrupting the passive layer and thereby reducing corrosion resistance^33^.

### SEM analysis

**Fig. 12:**
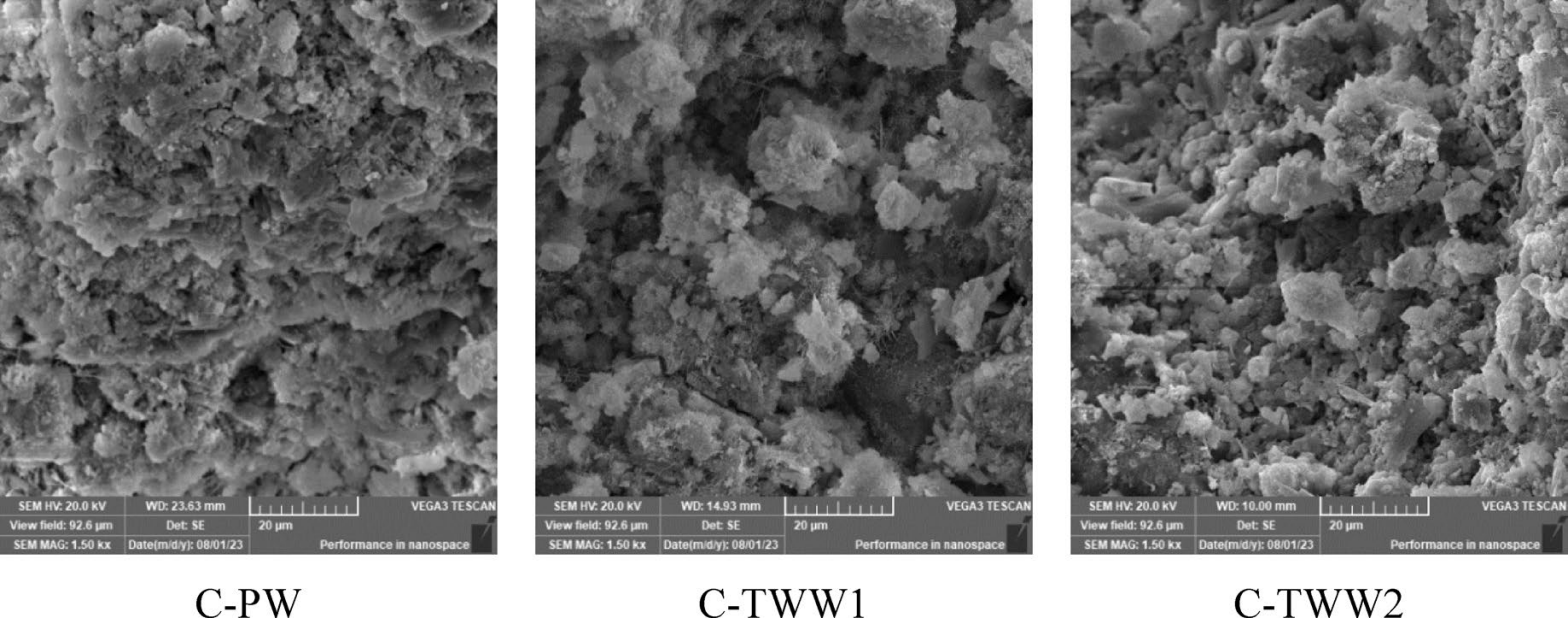
SEM analysis at 1500X magnification.

**Fig. 13:**
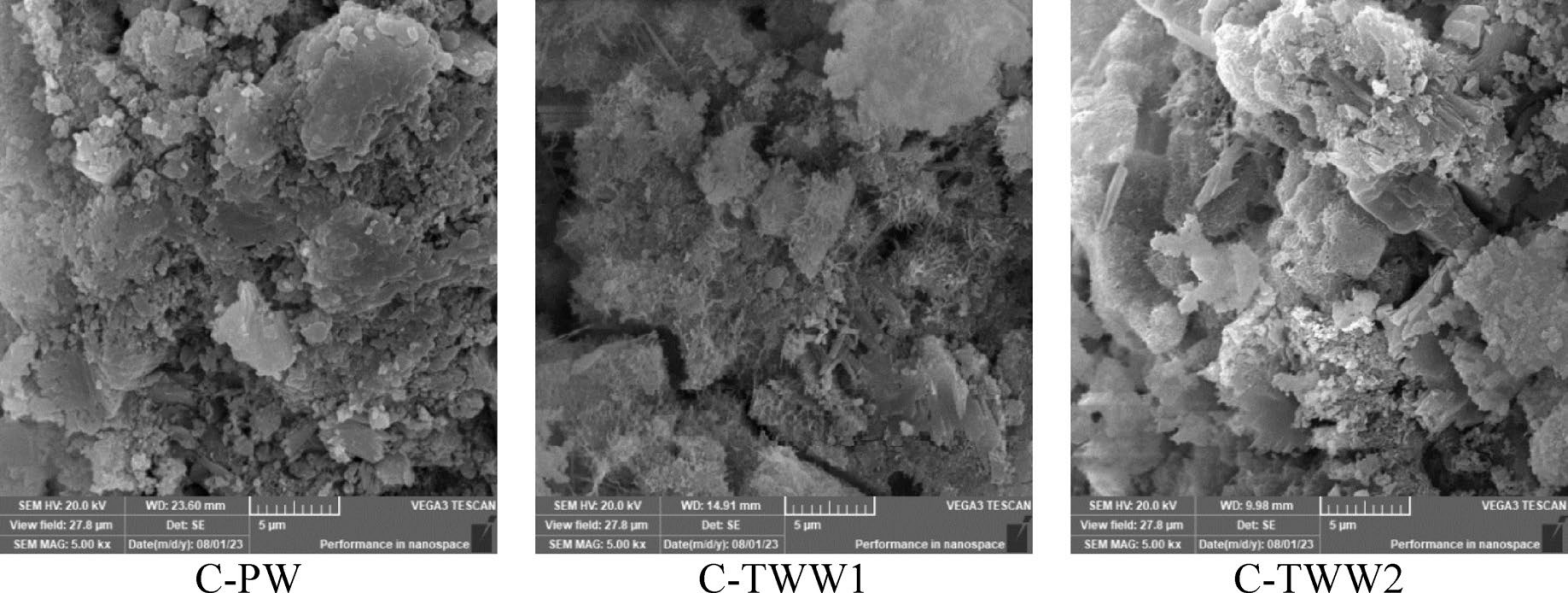
SEM analysis at 5000X magnification.

Figures 12 and 13 present the SEM results at magnifications of 1500× and 5000×, respectively. Results show that the C-PW sample exhibited well-stacked particles and a denser microstructure compared to C-TWW1 and C-TWW2. Moreover, C-TWW2 showed better crystal organization than C-TWW1, where noticeable pores and voids were observed in C-TWW1. This can explain the obtained results, where C-TWW2 showed better performance in terms of mechanical properties and durability. These results are consistent with previous literature findings, where larger voids and cracks were attributed to the presence of organic materials and impurities in TWW, which hinder the hydration process^10,25,34^.

## Conclusions and future recommendations

### Conclusions

Based on the experimentally obtained results, the detailed discussion of these results and the comparison of these results with the limits of different codes and standard specifications, the following points can be concluded:

- TWW reduced the workability of concrete, due to the presence of high amounts of fine solids compared to potable water.
- Although using TWW in mixing and curing concrete caused a noticeable reduction in compressive strength at early ages, the reduction in compressive strength became negligible at later ages.
- The impact of both types of TWW on durability related properties was not significant (i.e. water absorption, water penetration resistance, rapid chloride penetration, and sorptivity).
- TWW2 showed better results than TWW1 either in mechanical or durability related properties, which is attributed to the pore filling effect caused by high content of total dissolved solids in TWW2.
- TWW intensifies corrosion, as the mass loss due to corrosion increased in TWW1 and TWW2 by 63.2 and 57.4% respectively, indicating that TWW is more suitable for usage in unreinforced concrete.

### Future recommendations

Based on the results of this study, the following recommendations are suggested for future studies to fully understand the effect of TWW on concrete:

- The consistency in the properties of TWW at different seasons through the year should be studied to determine the range of variations in the properties of TWW collected from the same source. Moreover, it’s recommended that more than one sample should be tested from every water treatment plant.
- The pore size distribution should be studied on concrete mixed with TWW, as it will help in understanding the effect of TWW on the durability of concrete.

## Data Availability

The datasets generated during the current study are available from the corresponding author on reasonable request.
